# Identifying thresholds in the impacts of an invasive groundcover on native vegetation

**DOI:** 10.1038/s41598-021-98667-5

**Published:** 2021-10-15

**Authors:** Luke S. O’Loughlin, F. Dane Panetta, Ben Gooden

**Affiliations:** 1 Health and Biosecurity, Commonwealth Scientific and Industrial Research Organisation, Canberra, Australia; 2 The University of Melbourne, Melbourne, Australia

**Keywords:** Ecology, Community ecology, Invasive species

## Abstract

Impacts of invasive species are often difficult to quantify, meaning that many invaders are prioritised for management without robust, contextual evidence of impact. Most impact studies for invasive plants compare heavily invaded with non-invaded sites, revealing little about abundance–impact relationships. We examined effects of increasing cover and volume of the non-native herbaceous groundcover *Tradescantia fluminensis* on a temperate rainforest community of southern Australia. We hypothesised that there would be critical thresholds in *T. fluminensis* abundance, below which the native plant community would not be significantly impacted, but above which the community’s condition would degrade markedly. We modelled the abundance–impact relationship from 83 plots that varied in *T. fluminensis* abundance and landscape context and found the responses of almost all native plant indicators to invasion were non-linear. Native species richness, abundance and diversity exhibited negative exponential relationships with increasing *T. fluminensis* volume, but negative threshold relationships with increasing *T. fluminensis* cover. In the latter case, all metrics were relatively stable until cover reached between 20 and 30%, after which each decreased linearly, with a 50% decline occurring at 75–80% invader cover. Few growth forms (notably shrubs and climbers) exhibited such thresholds, with most exhibiting negative exponential relationships. *Tradescantia fluminensis* biomass increased dramatically at > 80% cover, with few native species able to persist at such high levels of invasion. Landscape context had almost no influence on native communities, or the abundance–impact relationships between *T. fluminensis* and the plant community metrics. Our results suggest that the diversity of native rainforest community can be maintained where *T. fluminensis* is present at moderate-to-low cover levels.

## Introduction

Invasive non-native plants negatively impact native species, communities and ecosystems^1,2^, yet the ecological consequences of many invaders remain under-quantified^3–5^. Reasons for this include both practical considerations, such as management action prioritisation under limited resources^6,7^ and research limitations, such as inconsistent definitions, study designs, and theories for understanding invasive species impacts^4,8,9^. Much empirical research on invasive plant impacts assesses only the largest effect of invasion by comparing “invaded” with “uninvaded control” areas, with fewer studies considering how impacts vary across a gradient of invader abundance^10–13^. Invader abundance–impact relationships may be linear or nonlinear and often vary according to the impact metric under consideration^11,14^. In some cases, a nonlinear relationship may reveal the existence of a cover-density threshold, beyond which there is a marked decrease in an impact metric with further increases in invasive species cover (e.g.^12^).

Invader abundance–impact relationships can be mediated by the functional traits and other characteristics of the resident native species that comprise the invaded community^10,15,16^. For example, Gooden et al.^12^ observed an impact threshold of approximately 85% cover for the invasive shrub *Lantana camara* on native vine species richness, but a significantly lower threshold for richness of native ferns (~ 30% cover), indicating differential resistance to invader impacts between resident species. Similarly, Fried and Panetta^17^ identified a lower impact threshold for the cover of a sub-component of the community (66% invader cover for perennial forbs) than for total vegetation cover (86%) in response to increasing abundance of the invasive shrub *Baccharis halimifolia*. Identifying plant growth form impact thresholds would elucidate which components of the recipient vegetation community are most severely impacted by non-native plant invasion and hence help to identify the species that are most likely to benefit from proactive management action^11,14^.

The functional response of native species to the altered conditions of an invaded ecosystem can also influence abundance–impact relationships. For example, shade-tolerant native species may benefit where an invader decreases light to the understorey, resulting in a functional shift in community composition without an overall reduction in vegetation diversity^18,19^. The abundance–impact relationship between an invasive plant and the functional diversity of a community may differ from the impact relationship with other more general indicators, such as native species richness^4,19,20^.

The abundance–impact relationship for any invasive species is likely context-specific, varying in space and time, and influenced by other habitat or landscape factors^4,21^. Landscape modification can drive invader impacts on native diversity by promoting non-native species abundance^21–23^ or amplifying invasive species’ per capita effects on native vegetation^24,25^. For example, in a study that controlled for the presence of disturbance and invasion, Sokol et al.^25^ found that logging plus invasion was associated with larger, smaller, or reversed impacts on different soil properties than simply invasion alone. Therefore, it is likely that landscape contextual factors (such as disturbance) would also modulate invader abundance–impact relationships, such that the magnitude of native diversity decline in response to invasion will be greatest in certain landscape contexts.

The aim of our study was to evaluate the abundance–impact relationships of the invasive ground-cover herb *Tradescantia fluminensis* with native temperate rainforest vegetation across variation in landscape context. Our study landscape comprised remnant cool temperate rainforest that occurred primarily in fragmented, often linear patches along streams and roadside reserves, intermixed with anthropogenic landscape features, such as suburban dwellings, footpaths, roads, industrial infrastructure and cleared pastures used for the cultivation of crops or livestock grazing. Our hypotheses were: (1) that there would be a critical threshold in the abundance–impact relationship between *T. fluminensis* and the native plant community, below which there would be no major impact of invasion; (2) that critical thresholds in the abundance–impact relationship of *T. fluminensis* would vary among different native plant growth forms; and (3) that abundance–impact relationships of *T. fluminensis* would be influenced by landscape context. We expected that a low-to-moderate cover of *T. fluminensis* would need to develop before there were major changes in plant diversity, but that this threshold would be: (1) lower for plant growth forms that most directly compete with *T. fluminensis* (such as native spreading herbs); (2) higher for plant growth forms that may have some resistance to invasion (such as climbers or tree ferns that can grow above *T. fluminensis*); and (3) lower in areas closer to modified landscape features (such as roads, footpaths, and urban gardens) that could amplify the impacts of *T. fluminensis*.

## Results

### Summary of vegetation composition

A total of 91 vascular plant taxa, representing 45 families, was recorded in this study. Of these predominantly perennial species (~ 95%), 61 were native (~ 68%), 28 were non-native (~ 31%) and two could not be identified to species level (species list provided in Appendix 3). Only five species were recorded from more than one-third of plots: *Tetrarrhena juncea* (graminoid; present in 54% of plots), *Australina pusilla* (forb; 54%), *Coprosma quadrifida* (shrub; 40%), *Hackelia latifolia* (herb; 37%) and *Polystichum proliferum* (fern; 35%). Most species (61%) were rarely encountered, occurring in fewer than 5% of plots (Appendix 3).

### Native species richness, relative abundance, and diversity

**Figure 1:**
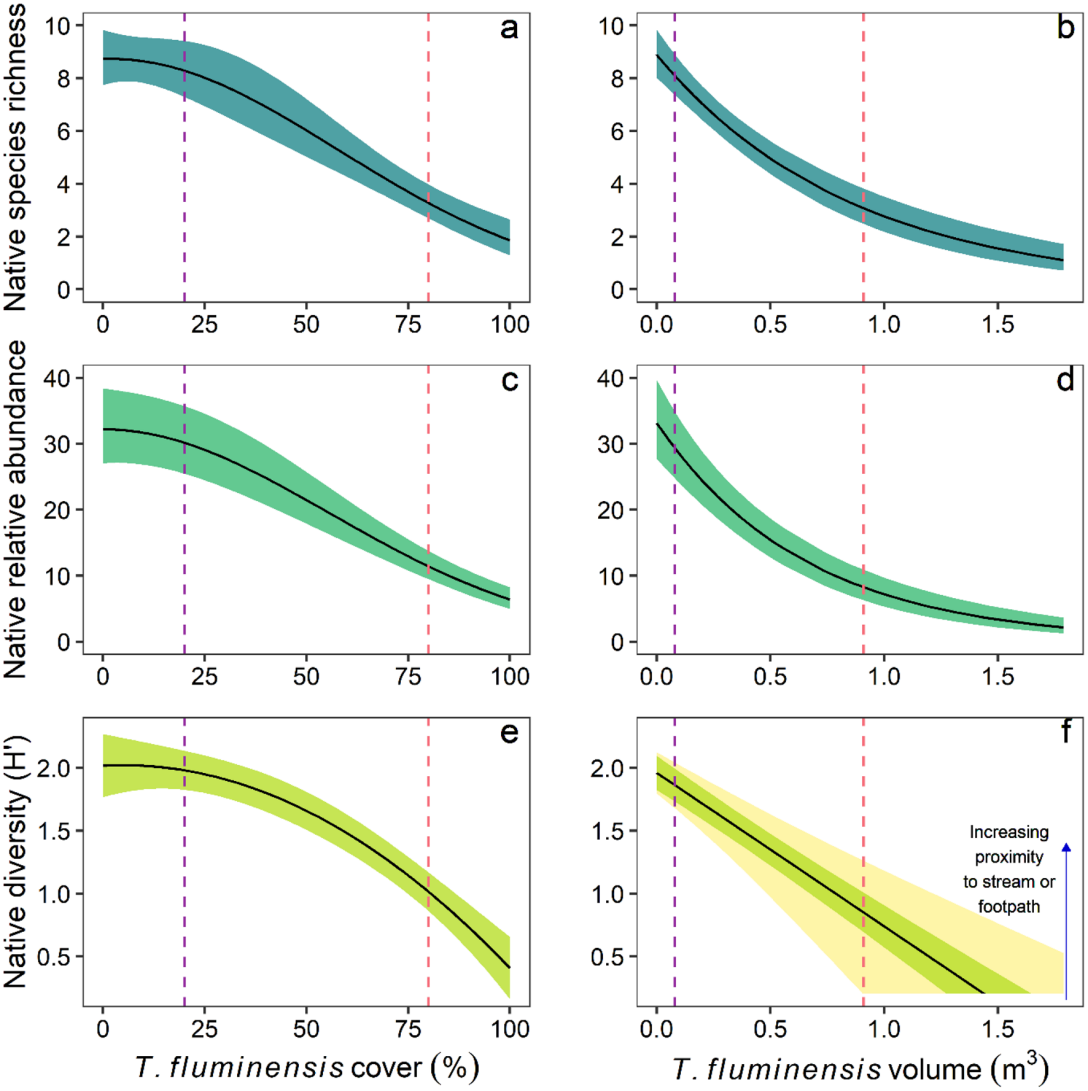
The response of native plant species richness (**a**,**b**), relative % foliage cover abundance (**c**,**d**), and diversity (Shannon–Wiener Diversity Index, H′) (**e**,**f**) to increasing foliage cover (%) and volume (m^3^) of *Tradescantia fluminensis*, measured in 2 m × 2 m quadrats (*n* = 83). Vertical dotted lines represent 20% (purple) and 80% (pink) cover values for *T. fluminensis*. Solid black lines represent predicted values from the top ranked GLMM (richness and abundance) or LMM (diversity), bound by 95% confidence intervals. These predictions are based on averaged predicted values from all top-ranked models (ΔAICc < 2), with other predictor variables held at their mean values. The exception to this is the predicted response of Diversity H′ to *T. fluminensis* volume (**f**), where there was a significant interactive effect of distance from streams and footpaths with *T. fluminensis*. Therefore, along with predictions where these two landscape covariates are held at their mean (i.e. the solid black line in plate **f**), we also included the predicted 95% confidence intervals (yellow), where the magnitude of native diversity H′ decline is either less severe with increasing proximity to streams or footpaths (as indicated) or more severe with greater distance from streams or footpaths.

*Tradescantia fluminensis* cover and volume featured in all top-ranked models for the three native plant response variables: species richness, relative vegetation abundance and diversity (H′). In all models, *T. fluminensis* was strongly negatively associated with each native plant response variable (Table 1). In response to increased *T. fluminensis* cover, native species richness, abundance and diversity (H′) were all relatively stable until *T. fluminensis* cover reached between 20 and 30%, after which the native variables decreased by 66–75% (Fig. 1a,c,e).

**Table 1:** Model results testing the effects of *Tradescantia fluminensis* foliage cover (%), volume (m^3^) and landscape predictor variables on native species richness, relative % foliage cover abundance and diversity (Shannon–Wiener Diversity Index, H′); *n* = 83.

Native response variable	Effect	Trad cover		Trad volume	
		Coefficient (95% CI)	Statistic	Coefficient (95% CI)	Statistic
Species richness	Intercept	1.72 (1.63, 1.83)	**32.89**	1.70 (1.56, 1.83)	**25.19**
	Trad (linear)	− 4.90 (− 5.98, − 3.82)	**8.86**	− 0.63 (− 0.81, − 0.45)	**6.80**
	Trad (quad.)	− 1.26 (− 2.16, − 0.36)	**2.74**		
	Dist. to stream	− 0.04 (− 0.13, 0.06)	0.75	− 0.09 (− 0.25, 0.06)	1.19
	Dist. to footpath			− 0.14 (− 0.31, 0.04)	1.50
	Dist. to forest edge			0.02 (− 0.06, 0.10)	0.46
	Trad × stream			− 0.14 (− 0.33, 0.05)	1.47
	Trad × footpath			− 0.24 (− 0.48, 0.00)	1.97
Relative abundance	Intercept	2.96 (2.79, 3.13)	**33.77**	2.87 (2.67, 3.06)	**29.32**
	Trad (linear)	− 5.48 (− 6.37, − 4.60)	**12.14**	− 0.80 (− 0.99, − 0.62)	**8.46**
	Trad (quad.)	− 1.64 (− 2.48, − 0.81)	**3.88**		
	Dist. to garden	− 0.02 (− 0.16, 0.05)	0.42	− 0.02 (− 0.21, 0.04)	0.42
	Dist. to forest edge	0.01 (− 0.10, 0.08)	0.10	0.01 (− 0.13, 0.06)	0.22
	Dist. to footpath			− 0.05 (− 0.30, 0.05)	0.55
	Dist. to road			− 0.24 (− 0.56, 0.07)	1.30
	Trad × footpath			− 0.06 (− 0.43, 0.05)	0.59
	Trad × road			− 0.34 (− 0.81, 0.11)	1.34
Diversity ( H′)	Intercept	1.51 (1.37, 1.64)	**22.07**	1.46 (1.31, 1.61)	**22.99**
	Trad (linear)	− 4.69 (− 5.43, − 3.96)	**12.51**	− 0.64 (− 0.75, − 0.55)	**− 14.11**
	Trad (quad.)	− 1.41 (− 2.16, − 0.67)	**3.71**		
	Dist. to stream	− 0.01 (− 0.15, 0.01)	0.31	− 0.16 (− 0.24, − 0.08)	**− 3.96**
	Dist. to footpath	− 0.00 (− 0.12, 0.07)	0.03	− 0.17 (− 0.27, − 0.08)	**− 3.35**
	Trad × stream	− 0.00 (− 0.14, 0.02)	− 0.08	− 0.17 (− 0.26, − 0.07)	**− 3.58**
	Trad × footpath			− 0.21 (− 0.34, − 0.07)	**− 3.09**

Standardised regression coefficients with 95% confidence intervals and test statistic (GLMM Z-value for species richness and relative abundance, LMM t-value for diversity) from model averaging are shown for predictor variables that featured in the top-ranked models (ΔAICc < 2) for each native response variable (see Table S2). Bold values denote significant effects (*P* < 0.05).

**Figure 2:**
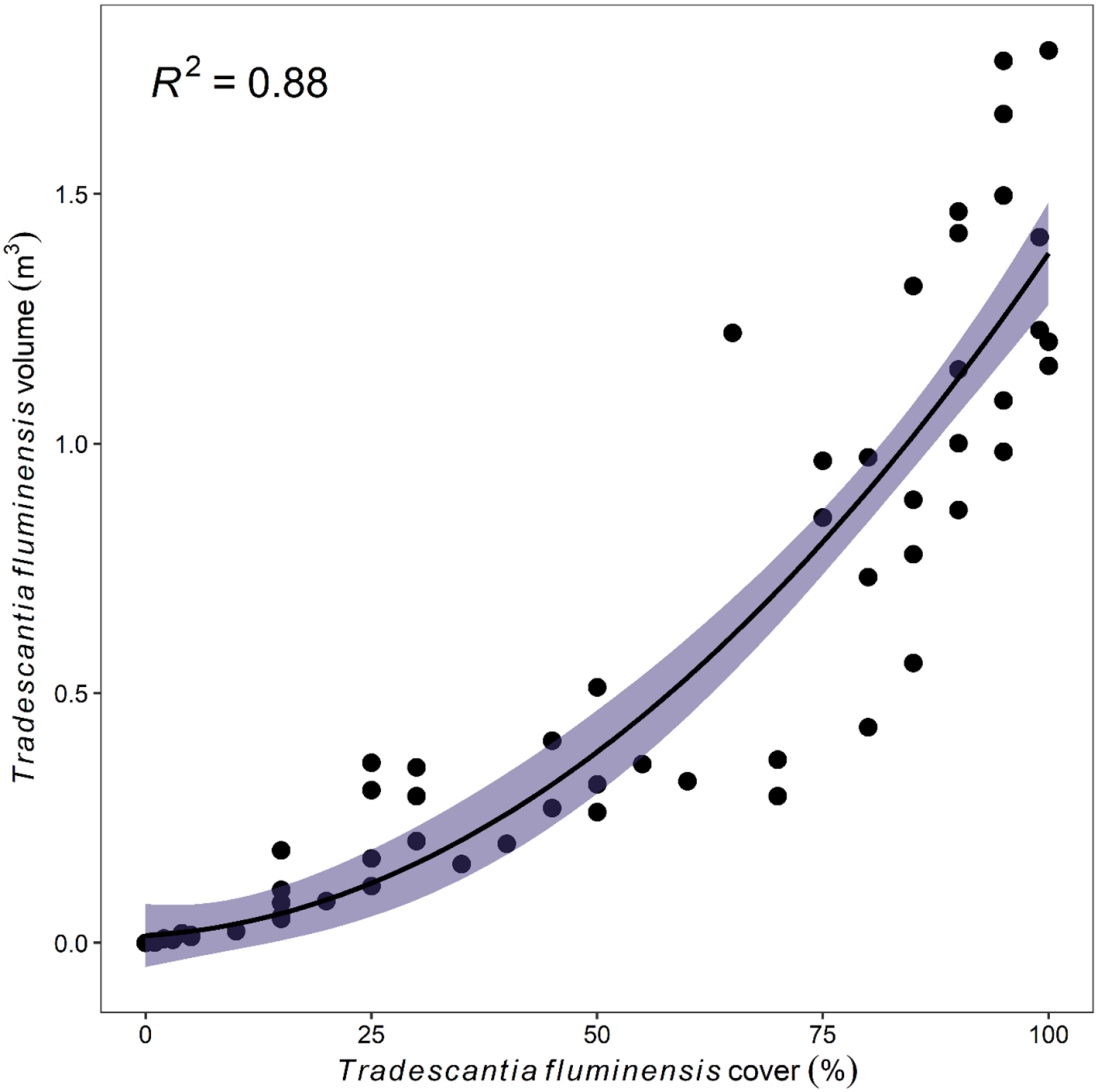
Relationship between *Tradescantia fluminensis* foliage cover (%) and volume (m^3^), measured in 2 m × 2 m quadrats. The solid line represents the fitted values from a polynomial regression (*n* = 83).

Native species richness and abundance also decreased significantly with increasing *T. fluminensis* volume, with a 50% decline in each occurring before *T. fluminensis* volume reached 0.5 m^3^ (Fig. 1b,d). This volume corresponded with approximately 80% cover (Fig. 2). For volume, the shape of each response curve was a negative exponential relationship. Native plant diversity (H′) decreased more linearly with increasing *T. fluminensis* volume, but with greater variability in the response owing to significant interactive effects with distance to streams and footpaths (Table 1, Fig. 1f)—i.e. the magnitude of decline in diversity (H′) in response to increasing *T. fluminensis* volume was greatest further away from streams and footpaths. No other landscape co-variates directly influenced any native variables (Table 1).

Non-native plants species richness was not associated with *T. fluminensis* cover (Coeff. = − 0.23, 95% CI [− 0.73, 0.14]) but was negatively associated with *T. fluminensis* volume (Coeff. − 0.52, % 95 CI [− 0.82, − 0.22]) based on model averaging of top-ranked models.

### Species richness and abundance of native plant growth forms

**Figure 3:**
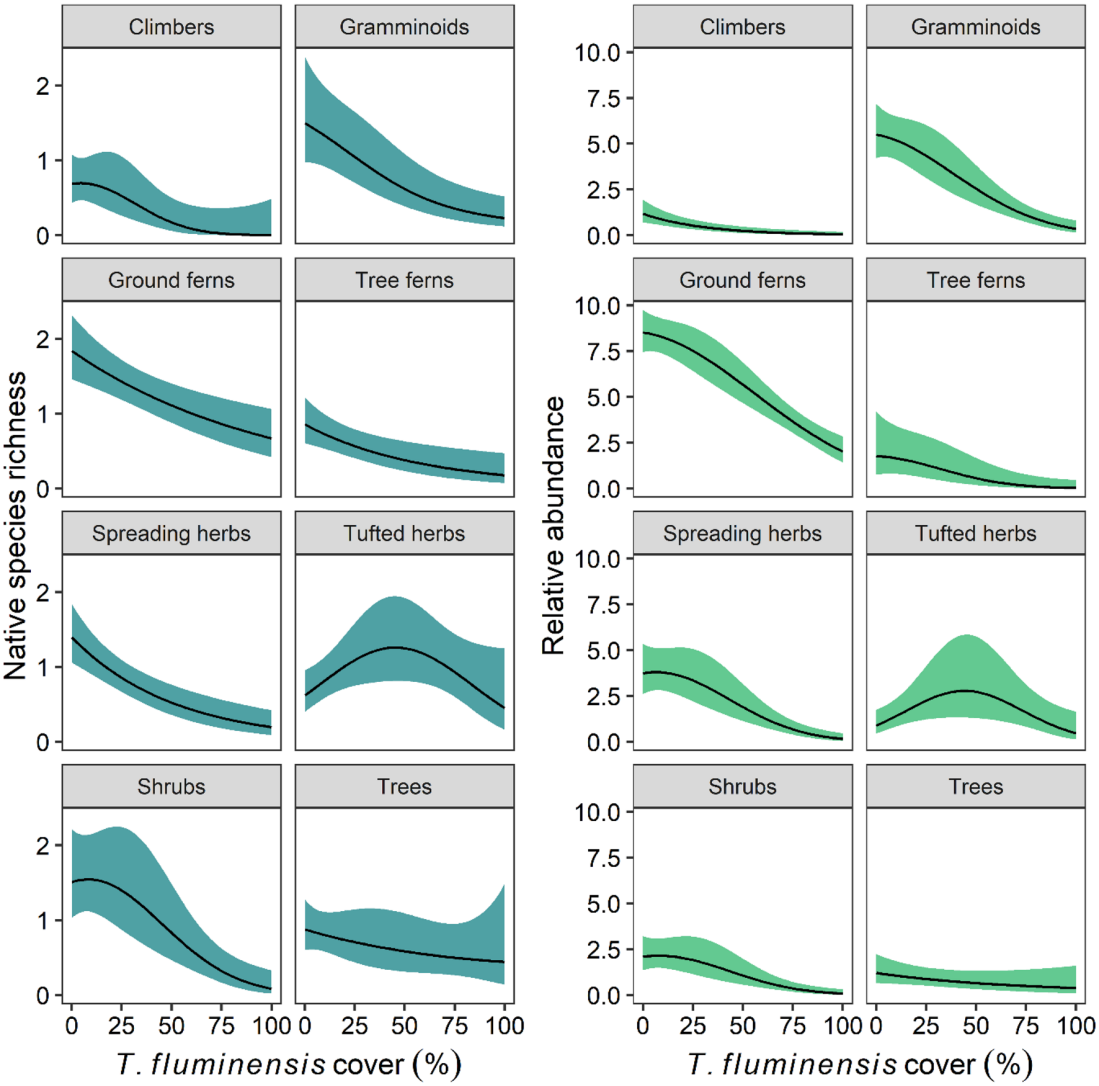
The response of native species richness and relative % foliage cover abundance of different plant functional growth forms to increasing % foliage cover of *Tradescantia fluminensis*, measured in 2 m × 2 m quadrats (*n* = 83). Solid black lines represent predicted values from the top-ranked GLMM, bound by 95% confidence intervals. These predictions are based on averaged predicted values from all top-ranked model (ΔAICc < 2), with other predictor variables held at their mean values.

*Tradescantia fluminensis* cover featured in all top-ranking models for the species richness and cover abundance of the eight growth form groups. In all cases, except for native trees, increased *T. fluminensis* cover was negatively associated with growth-form species richness and abundance (Table 2, Fig. 3). On average, across the gradient of *T. fluminensis* cover, the species richness of graminoids, ground ferns, tree ferns, and spreading herbs decreased exponentially by > 50% (i.e. from approximately 1–2 to 0–1 species per plot, Fig. 3). The species richness of climbers and shrubs displayed a negative threshold response, only decreasing significantly after approximately 20% *T. fluminensis* cover was reached (Fig. 3) and approaching zero species on average per plot after *T. fluminensis* cover exceeded 75%.

**Table 2:** Model results testing the effects of *Tradescantia fluminensis* foliage cover (%), volume (m^3^) and landscape predictor variables on native species richness and relative % foliage cover abundance of native plant growth forms; *n* = 83.

Native growth form	Effect	Species richness		Relative abundance		
		Coefficient (95% CI)	Z	Coefficient (95% CI)		Z
Climbers	Intercept	− 2.07 (− 3.51, − 0.63)	**2.81**	− 1.52 (− 2.17, − 0.87)		**4.56**
	Trad (linear)	− 19.34 (− 36.94, − 1.72)	**2.15**	− 1.29 (− 2.02, − 0.56)		**3.45**
	Trad (quad.)	− 5.21 (− 13.16, 2.73)	1.29			
	Dist. to forest edge	0.03 (− 0.08, 0.38)	0.35	0.02 (− 0.10, 0.37)		0.32
	Dist. to garden	0.06 (− 0.11, 0.52)	0.48	0.06 (− 0.10, 0.50)		0.49
	Dist. to road	0.06 (− 0.64, 0.19)	0.42	− 0.04 (− 0.55, 0.21)		0.35
	Dist. to footpath	0.01 (− 0.27, 0.46)	0.15	0.01 (− 0.21, 0.42)		0.20
Graminoids	Intercept	− 0.24 (− 0.51, 0.02)	**1.80**	0.84 (0.58, 1.09)		**6.33**
	Trad (linear)	− 0.53(− 0.83, − 0.24)	**− 3.57**	− 9.10 (− 11.73, − 6.47)		**6.78**
	Trad (quad.)			− 1.92 (− 4.00, 0.17)		1.80
	Dist. to footpath	0.03(− 0.10, 0.32)	0.43	0.06 (− 0.06, 0.33)		0.60
	Dist. to forest edge	− 0.01(− 0.28, 0.17)	0.19			
	Dist. to stream			0.36 (0.14, 0.58)		**3.27**
	Trad × stream			0.34 (0.12, 0.56)		**3.03**
Ground ferns	Intercept	0.23 (0.02, 0.42)	**2.28**	1.69 (1.56, 1.82)		**25.04**
	Trad (linear)	− 0.39(− 0.61, − 0.18)	**− 3.53**	− 4.95 (− 6.42, − 3.48)		**6.62**
	Trad (quad.)			− 1.09 (− 2.08, − 0.12)		**2.18**
	Dist. to garden			− 0.16 (− 0.35, 0.02)		1.69
	Dist. to stream			− 0.05 (− 0.01, 0.16)		0.92
	Dist. to footpath			− 0.01 (− 0.16, 0.10)		0.22
	Dist. to road			− 0.06 (− 0.05, − 0.15)		0.28
	Trad × garden			− 0.07 (− 0.44, 0.38)		0.56
	Trad × footpath			− 0.03 (− 0.32, 0.01)		0.43
	Trad × stream			− 0.01 (− 0.19, 0.06)		0.22
Tree ferns	Intercept	− 0.74 (− 1.24, − 0.23)	**2.87**	− 0.79 (− 1.70, 0.12)		1.70
	Trad (linear)	− 0.59 (− 1.16, − 0.02)	**2.03**	− 13.50 (− 22.2, − 4.8)		**3.03**
	Trad (quad.)			− 3.64 (− 7.91, − 0.63)		1.67
	Dist. to garden	− 0.17 (− 1.71, 0.16)	0.43	− 1.13 (− 2.40, 0.07)		1.74
	Dist. to stream	− 0.06 (− 0.51, 0.15)	0.46	− 0.13 (− 0.71, 0.11)		0.66
	Dist. to road	− 0.22 (− 1.01, 0.21)	0.71	− 0.47 (− 1.14, − 0.01)		1.35
	Dist. to footpath			− 0.07 (− 0.75, 0.21)		0.42
	Dist. to forest edge			0.01 (0.40, 0.79)		0.15
	Trad × road	− 0.06 (− 1.35, 0.47)	0.27			
	Trad × garden	− 0.19 (− 1.88, 0.17)	0.44	− 1.75 (− 3.17, − 0.44)		**2.34**
Spreading herbs	Intercept	− 0.40 (− 0.70, − 0.10)	**2.59**	0.45 (0.11, 0.79)		**2.64**
	Trad (linear)	− 0.75 (− 1.09, − 0.41)	**4.31**	− 10.00 (− 13.29, − 6.71)		**5.95**
	Trad (quad.)			− 2.85 (− 5.49, − 0.20)		**2.11**
	Dist. to footpath	− 0.02 (− 0.38, 0.15)	0.31	− 0.05 (− 0.44, 0.10)		0.46
	Dist. to forest edge	− 0.01 (− 0.31, 0.15)	0.25	− 0.01 (− 0.30, 0.17)		0.19
	Dist. to garden	− 0.01 (− 0.29, 0.18)	0.18	− 0.02 (− 0.35, 0.13)		0.29
Tufted herbs	Intercept	− 0.29 (− 0.60, 0.02)	1.86	0.01 (− 0.53, 0.56)		0.04
	Trad (linear)	− 0.11 (− 3.26, 3.05)	0.07	− 0.72 (− 5.21, − 3.78)		0.31
	Trad (quad.)	− 2.68 (− 5.26, − 0.10)	**2.04**	− 5.17 (− 9.03, − 1.32)		**2.63**
	Dist. to road	0.28 (0.01, 0.56)	**2.04**	0.46 (0.04, 0.88)		**2.16**
	Dist. to garden	0.23 (− 0.09, 0.55)	1.42	0.16 (− 0.20, 1.06)		0.56
	Dist. to stream	− 0.03 (− 0.45, 0.18)	0.30	− 0.17 (− 0.79, 0.14)		0.72
	Dist. to footpath	− 0.01 (− 0.15, 0.29)	0.23			
	Dist. to forest edge			− 0.17 (− 1.24, 0.24)		0.53
	Trad × garden	0.36 (− 0.08, 0.80)	1.61	0.09 (− 0.16, − 1.37)		0.35
	Trad × road	0.02 (− 0.29, 0.51)	0.19			
Shrubs	Intercept	− 0.53 (− 0.89, − 0.18)	**2.97**	− 0.19 (− 0.63, 0.24)		0.89
	Trad (linear)	− 7.75 (− 11.81, − 3.69)	**3.74**	− 10.58 (− 14.98, − 6.18)		**4.71**
	Trad (quad.)	− 2.27 (− 5.11, 0.56)	1.57	− 3.18 (− 6.52, 0.15)		1.87
	Dist. to road	0.04 (− 0.09, 0.35)	0.46	0.24 (− 0.07, 0.66)		0.23
	Dist. to footpath	− 0.02 (− 0.38, 0.15)	0.28			
	Dist. to stream	− 0.01 (− 0.32, 0.19)	0.17	− 0.04 (− 0.52, 0.16)		0.72
	Dist. to garden	− 0.02 (− 0.47, 0.12)	0.28	− 0.09 (− 0.59, 0.09)		0.57
	Dist. to forest edge			− 0.01 (− 0.41, 0.21)		0.88
	Trad × road			0.02 (− 0.43, 0.74)		0.86
Trees	Intercept	− 0.44 (− 0.84, − 0.04)	**2.18**	− 0.33 (− 1.03, 0.37)		0.90
	Trad (linear)	− 0.32 (− 0.74, 0.16)	1.29	− 0.47 (− 1.22, 0.24)		0.19
	Dist. to road	− 0.38 (− 0.97, 0.09)	1.32	− 0.59 (− 1.32, 0.04)		0.12
	Dist. to forest edge	0.33 (− 0.49, 1.85)	0.62	0.57 (− 0.65, 2.53)		0.47
	Dist. to garden	0.01 (− 0.17, 0.40)	0.23	− 0.09 (− 1.35, 0.43)		0.74
	Dist. to stream			0.01 (− 0.29, 0.55)		0.88
	Trad × forest edge	0.28 (− 0.13, 1.96)	0.54	0.34 (− 0.38, 2.68)		0.62
	Trad × road	− 0.03 (− 1.09, 0.39)	0.22			

Standardised regression coefficients with 95% confidence intervals and test statistic (GLMM Z-value for species richness and relative abundance, LMM t-value for diversity) from model averaging are shown for predictor variables that featured in the top-ranked models (ΔAICc < 2) for each native response variable (see Table S2). Bold values denote significant effects (*P* < 0.05).

The relative abundance of graminoids, ground ferns, spreading herbs and shrubs was relatively stable until *T. fluminensis* cover reached approximately 20%, after which it decreased significantly (Fig. 3). The species richness and relative abundance of tufted herbs were predicted to be highest at moderate levels of *T. fluminensis* cover (~ 50%) and lowest where *T. fluminensis* was either absent or dominant (Fig. 3). The richness and relative abundance of tufted herbs were also positively associated with increased distance from a road, and the relative abundance of graminoids was positively associated with increased distance from a stream (with a significant *T. fluminensis* by distance from stream interaction) (Table 2). No other significant effects of landscape factors were identified (Table 2).

## Discussion

### Importance of the invader abundance metric for understanding thresholds of impact on native communities

We found that invasion of *T. fluminensis* was strongly negatively associated with the diversity of the native cool temperate rainforest community. Native species richness, abundance and diversity all decreased to close to zero as *T. fluminensis* foliage increased to 100% cover. Such patterns are consistent with the general negative effects of invasive non-native plants on recipient native plant communities^1,2^, and particularly ground-cover invaders that have been demonstrated to competitively exclude and suppress native species^20,26,27^.

In correlative (non-manipulative) studies like ours, negative associations between invader abundance and the characteristics of native vegetation communities can arise from either the direct impacts of invasion on the resident community (where a reduction in native species richness is a *consequence* of invasion) or via an indirect mechanism, whereby some form(s) of primary vegetation disturbance precipitates native species decline and commensurate increase in invader abundance. Such ‘driver-passenger’ models have been explored extensively in the literature (e.g.^28,29^) and are usually tested using small-scale manipulative experiments (e.g. invader addition or invader removal studies^16,30^) or rarely with longitudinal observations (e.g.^31^). However, such experiments usually lack context at a landscape scale and are thus unable to evaluate interactive effects of invasion and large-scale disturbances on resident native communities. Observational studies like ours, undertaken at a landscape scale, are critical in contextualising broad patterns of community change in association with non-native plant invasion. In our case, we ensured that the spectrum of *T. fluminensis* invasion was well-represented across a gradient of landscape disturbance, and such disturbance was shown to have very little negative effect on native vegetation diversity (discussed below). The strongest predictor of diversity decline was invader cover, thus indicating that *T. fluminensis* invasion is driving community change. This is consistent with observations that *T. fluminensis* can actively invade deeply shaded, healthy, intact rainforest stands, often at great distance from forest edges.

We identified a clear and consistent impact threshold of around 20% cover abundance of *T. fluminensis*, below which native species richness, abundance and diversity were relatively stable but above which they all declined linearly on average by approximately 80% as *T. fluminensis* cover approached 100%. This threshold based on *T. fluminensis* cover is markedly different from both generalised abundance–impact relationships quantified among invaders as a group and those of other invasive plants. A meta-analysis of abundance–impact relationships for all invasive species (plants and animals) by Bradley et al.^10^ revealed predominately linear relationships, and no density-dependence thresholds for impacts of invasive plants on native plants. Empirical tests of abundance–impact relationships for invasive plants vary considerably, from having no relationship with native species^32^, linear declines^17^, or much higher thresholds of cover before native species are impacted, such as ~ 70% for *Delairea ordorata*^33^, 75–80% for *Lantana camara*^12^ and ~ 80% for *Baccharis halimifolia*^17^. The 20% cover threshold we identified is also significantly different from the abundance–impact relationships previously determined for *T. fluminensis* by McAlpine et al.^27^ and Standish et al.^34^. First, our threshold was much lower than the ~ 70 to 80% volume threshold estimated by McAlpine et al.^27^. This difference potentially reflects greater resistance of the native plant community to the impacts of *T. fluminensis* in their New Zealand study system. Conversely, Standish et al.^34^ did not identify any impact threshold in native species richness or seedling density, both of which decreased exponentially with increasing *T. fluminensis* biomass.

We found that *T. fluminensis* cover and volume scaled positively but non-linearly, since infestations tended to rapidly get deeper with increasing cover. This resulted in no identifiable threshold when considering *T. fluminensis* volume, with native richness, abundance and diversity all decreasing by around 50% from 0 to 0.75 m^3^. Indeed, we detected negative exponential declines in native species indicators with increasing *T. fluminensis* volume. In contrast, McAlpine et al.^27^ found a volume impact threshold of around 0.75–1.00 m^3^ of *T. fluminensis*, below which native species richness and abundance did not decline. Our results highlight that invasion abundance–impacts accumulate differently over a gradient of invasion depending on which metric of invader abundance (in our cases, cover versus volume) is modelled.

### Non-native plant impact thresholds on native vegetation do not vary across gradients of anthropogenic landscape modification

Landscape context had limited influence on the abundance–impact relationships between *T. fluminensis* and native species richness, abundance, and diversity. Although all landscape factors we measured featured in supported models at least once, most had no significant effect on native response variables. Distance to stream, footpath and forest edge were the most common factors that contributed to explaining some variation in the native plant metrics, without themselves having significant main or interactive effects with *T. fluminensis* invasion. The one exception was the relationship between *T. fluminensis* volume and native diversity. We found that the magnitude of reduction in native diversity in response to *T. fluminensis* invasion (based on invader volume) increased with distance from a stream or footpath. The specific mechanisms underpinning this interactive effect are unclear. Stream embankments infested with shallow-rooted *T. fluminensis* plants are regularly disturbed by floodwater, which can rapidly dislodge large *T. fluminensis* mats that raft downstream (Gooden Pers. Obs.), whilst native species, particularly those with deeper root networks, may be either relatively less disturbed by flooding or can rapidly revegetate denuded soil. We suggest that the proximity to streams, and commensurate disturbance by floodwater, may possibly ameliorate (but not completely offset or reverse) the adverse effects of dense *T. fluminensis* infestations on native diversity.

Despite other studies finding strong evidence that invader impacts are amplified where disturbance is higher^24,25,35^, we did not observe more severe impacts of *T. fluminensis* on native species when closer to roads or the forest–urban interface. This may result from the surveys being undertaken in a highly human-modified landscape, with remnant native forests restricted in many cases to linear strips along stream embankments within a suburban-agricultural matrix. Consequently, the pool of resident native species may already have been significantly reduced across the landscape prior to *T. fluminensis* invasion, thus resulting in the null effect of contemporary landscape variables on most native vegetation diversity. Nevertheless, our results show that the strongest current driver of local vegetation diversity, independent of landscape context, is proliferation of a non-native plant invader.

### Comparing abundance–impact relationships: whole community versus growth form metrics

A major aim of this study was to determine the abundance–impact relationships between *T. fluminensis* and native species growth forms. Overall, there was discordance between the abundance–impact relationships apparent for whole community metrics and those for plant growth forms. When *T. fluminensis* abundance was assessed as percentage foliar cover, impact thresholds were observed for all three community metrics. For growth forms, however, impact thresholds in species richness were detectable only for climbers and shrubs. In terms of relative abundance, a threshold abundance–impact relationship was detectable for shrubs and spreading herbs, but no longer for climbers. These results could reflect differences in native species’ responses to non-native plant invasion arising from their intrinsic functional traits and highlight the importance of considering context-specific patterns of community change when quantifying the impacts of non-native plant invasion on recipient ecosystems (see related discussion in^36,37^). Alternatively, they could be an artefact of the low representation of species from each growth form in individual plots, such that invader abundance–impact thresholds become apparent when the data for all growth forms are aggregated at the community level.

Maximum species richness and relative abundance of tufted herbs at moderate cover of *T. fluminensis* (40–50% cover; Fig. 3) were unexpected results. This pattern suggests that tufted herbs may be naturally outcompeted by other native species in this community and initially benefit from *T. fluminensis* invasion before decreasing again as invader cover increases beyond 50%. The positive association with invasion could be due to either the decreased abundance of competitively dominant natives, the altered structural and abiotic conditions created by invasion (e.g. lower light^34^), or a coincidental response or tufted herbs to the same environmental conditions that promote *T. fluminensis* invasion. While there are some examples of native plants benefiting from invasive plants (e.g.^38^) and positive ecological effects more generally from invasion (e.g.^39^), a humped abundance–impact relationship is rarely hypothesised (i.e. not presented as a potential relationship in Bradley et al.^10^) and is rarely empirically demonstrated. The lack of discussion around “intermediate invasion” is interesting given that the humped-back diversity–productivity^40^ and diversity–disturbance^41^ relationships are important foundation theories of community ecology.

### Conclusions and management implications

The form of the abundance–impact relationship has important implications for the management of an invasive plant species^11,14,15,42^. Where the abundance–impact relationship is non-linear and an impact threshold can be identified, the appropriate management goal would be to maintain invader abundance below it (so-called “maintenance control”^11^). However, we have shown that for some growth forms the abundance–impact relationship for *T. fluminensis* is either exponential or linear, meaning that these plants are negatively impacted at low invasive plant cover values. To conserve these species, the “action threshold” (i.e., the target abundance of the invader at which management intervention is triggered) for the control of *T. fluminensis* would likely be lower than the impact threshold assessed in terms of less sensitive community metrics, such as native species richness, relative abundance, and diversity.

Herbicide application is considered the only practical way to control large infestations of *T. fluminensis*^43^. However, in Australia some of the herbicides registered for its control cannot be used close to waterways. Smaller infestations can be hand pulled, raked and rolled-up for manual removal, but plants will regrow from any remaining stem or stem fragments, meaning that control of this species is currently a protracted and labour-intensive activity. Biological control is an emerging management option that could potentially provide effective, sustainable control of *T. fluminensis* in Australia^44,45^*.* Given the community resistance to low-density invasion we observed, indications are that the recent introduction of a fungal biological control agent (*Kordyana brasiliensis*) could be highly beneficial to the native plant community should it prove effective at maintaining *T. fluminensis* at low cover levels.

With a well-established, widespread invader, such as *T. fluminensis*, a range of invasion statuses will exist in invasible plant communities, from uninvaded sites to those that are heavily invaded, with depauperate native diversity. In the context of scant available resources for active management intervention, there would presumably be required some a priori triaging of sites by some index of conservation value to decide where to maintain *T. fluminensis* cover at low levels. This would allow practitioners to aim for near-complete removal (and ongoing suppression of invasion) of *T. fluminensis* in areas identified as high conservation value, such as stands of cool temperate rainforest that contain threatened species. Further research is required to determine the prevalence of invasive plant impact thresholds and, where such abundance–impact relationships exist, to resolve the issue of whether to manage invasive plants according to community impact metrics or according to impacts of the invader on the community’s least resistant growth form(s).

## Materials and methods

Authors declare that this manuscript complies with all the institutional, national, and international guidelines and legislation, regarding all species and habitats studied.

### Study system

*Tradescantia fluminensis* Vell. (family Commelinaceae, commonly known as small-leaf spiderwort) is a sprawling herb native to rainforests of south-eastern Brazil^43^. Adventitious roots forming along stem nodes enable rapid clonal spread, including from fragmented stems^43^. *Tradescantia fluminensis* is considered a significant invasive plant of subtropical to cool temperate rainforest ecosystems worldwide, especially in New Zealand and Australia^46^, where it was introduced as a popular ornamental house and garden plant^43^. Naturalisation in native forests likely resulted from clonal propagation from adjacent suburban gardens, as well as from illegal dumping of garden waste^47^. In New Zealand, *T. fluminensis* can reduce the abundance and richness of native vegetation^27,34^, disrupt ecosystem processes, such as leaf litter decomposition rates and soil nutrients^48^, and native faunal assemblages (e.g. invertebrates^48,49^). Effects of *T. fluminensis* on Australian forest ecosystems are poorly understood.

Our study investigated the effects of *T. fluminensis* invasion on native plant community composition in the Dandenong Ranges, approximately 40 km east of Melbourne (Victoria), Australia (map provided in Appendix 1). The Dandenong Ranges has a moist temperate climate with mean annual rainfall of ~ 1,000 mm (Monbulk weather station^50^) and mean minimum and maximum daytime temperatures of 9.8 °C (austral winter, July) and 24.5 °C (austral summer, January), respectively (Ferny Creek weather station, Bureau of Meteorology 2020).

Plant communities across the Dandenong Ranges are characterised by a mix of tall wet eucalypt forests (dominated by *Eucalyptus regnans, E. obliqua* and *E. cypellocarpa*, with a dense understorey of mesophyllous rainforest trees, shrubs, herbs and ferns) and cool temperate rainforests (characterised by *Acacia melanoxylon*, *Atherosperma moschatum* and the tree ferns *Cyathea* spp. and *Dicksonia australis*), located along streamlines in moist gullies^51^. Except for several large reserves located within the Dandenong Ranges National Park (e.g. Ferntree Gully and Sherbrooke Forest; Incoll et al.^52^), remnant cool temperate rainforest occurs in fragmented, often linear patches along streams and roadside reserves intermixed with anthropogenic landscape features, such as suburban dwellings, footpaths, roads, industrial infrastructure and cleared pastures used for cultivation of crops or livestock grazing. Recent surveys by Incoll et al.^52^ identified *T. fluminensis* as a high-priority invasive plant for management across the Dandenong Ranges, due to its widespread distribution and ability to invade deeply shaded, cool temperate rainforest habitats without primary disturbance, where it can dominate the understorey vegetation and suppress the growth of native flora (see example photographs of *T. fluminensis* infestations in Appendix 2).

### Survey design and vegetation sampling

To assess the interactive effects of variation in *T. fluminensis* abundance and landscape context, our monitoring plots needed to be stratified in two ways. First, plots needed to represent the full gradient of invader abundance (0–100% cover) relatively evenly (i.e. approximately equal representation of no, low, medium, and high cover plots). Second, that gradient of invasion needed to be replicated relatively evenly among different landscape contexts, from highly modified to largely intact remnant vegetation. This stratification was achieved by targeted plot selection in the field. Across the study landscape (Appendix 1), eight different areas of streamside native cool temperate rainforest remnants were selected. Six of these areas were highly variable in their landscape context, with part of the stream being close to industrial or high-density urbanisation, some parts being close to parks or other semi-natural lands, and other parts being close to other remnant vegetation (wet sclerophyll forest) (Table 3; Appendix 1). The other two areas were selected because they were comparatively less modified and represented the best examples of undisturbed remnant vegetation (Table 3). Within each area, plots were selected at locations of no (0%), low (~ 1 to 33%), moderate (~ 34 to 66%), and high (~ 67 to 100%) *T. fluminensis* foliage cover within different landscape contexts. For example, if one part of a remnant area was near to a road and urban activity, and a separate part was close to a cultivated paddock, then we would aim to establish plots representing the complete gradient of *T. fluminensis* cover in each of those two different parts. This targeted plot selection ensured a relatively even representation of invasion level within different qualitative assessments of landscape context. The level of replication within each area varied based on the size of the area and status of *T. fluminensis*, but the total replication among plots of no, low and high *T. fluminensis* cover was close to even (Table 3).

**Table 3:** Total number of sites representing varying levels of *Tradescantia fluminensis* foliage cover (%) as identified across the eight different landscape areas.

Landscape area	*Tradescantia fluminensis* foliage cover (%)			
	Absent (0)	Low (~ 1 to 33)	Mod. (~ 34 to 66)	High (~ 67 to 100)
**Variable landscape context**				
Emerald Creek	1	0	1	3
Monbulk Creek-Birdsland	0	2	1	3
Monbulk Creek-National Road	1	4	2	3
Sassafras Creek	12	7	1	15
Sherbrooke Forest-East	3	0	0	0
Sayers Road	0	1	1	1
**Low modification landscape context**				
Clematis Creek-Monbulk Road	9	5	1	1
Kokoda Memorial Walk	0	3	2	0
Total	26	22	9	26

A total of 83 monitoring plots (2 × 2 m) was established across our study landscape following this targeted selection approach. This plot size has been shown previously to be a suitable scale to assess the responses of diverse ground-layer forest vegetation to invasion by herbaceous non-native scramblers (e.g. *T. fluminensis*) in New Zealand^27^ and grasses (e.g. the stoloniferous grass *Stenotaphrum secundatum*) in Australia^53^. In each 4 m^2^ plot, we visually estimated *T. fluminensis* percentage foliage cover to the nearest 5%. We also calculated the volume (m^3^) of space within the ground-layer vegetation occupied by *T. fluminensis* by multiplying average standing height (m; calculated from 10 random points across the plot as per Standish et al.^34^) by planar foliage cover (converted from % to m^2^), as a surrogate measure of biomass. Indeed, Standish et al.^34^ found that this measure of *T. fluminensis* volume was a very strong positive predictor of biomass (g. m^−2^). While plots were initially selected based on a quick categorical identification of *T. fluminensis* cover, we quantified cover and volume in this way so “invasion” could be considered a continuous variable in our analyses (Table 4).

**Table 4:** Explanatory variables used in the analysis.

Variable	Description	Range	Type
**Fixed effects**			
Trad cover (%)	Visually estimated cover of live *T. fluminensis* per plot	0–100 (median = 25)	Integer
Trad volume (m^3^)	Average standing height multiplied by foliage cover of *T. fluminensis*	0–1.79 (median = 0.17)	Numerical
Distance to forest edge (m)	Minimum linear distance to an edge of the forest remnant (measured remotely)	2–803 (median = 50)	Integer
Distance to stream (m)	Minimum linear distance to a stream, a potential source of *T. fluminensis* propagules (measured in the field)	0–82 (median = 5)	Integer
Distance to road (m)	Minimum linear distance to a road, an indicator of relative proximity to urban disturbance (measured remotely)	5–821 (median = 50)	Integer
Distance to footpath (m)	Minimum linear distance to a pedestrian footpath, an indicator of relative proximity to urban disturbance (measured remotely)	1–222 (median = 17)	Integer
Distance to garden (m)	Minimum linear distance to a road, an indicator of relative proximity to urban disturbance and potential source of *T. fluminensis* propagules (measured remotely)	5–951 (median = 86)	Integer
**Random effects**			
“Area”: to account for spatial autocorrelation	Landscape area identifier that represents the spatial clustering of plots along different streams (see Table 1)	–	Factor (8 levels)

We recorded the identity and origin (native or non-native) of all resident plant species rooted within and overhanging each 4 m^2^ plot. We only sampled vegetation growing within the ground and shrub layers (to a height of ≤ 2 m), which is likely to interact directly and most strongly with *T. fluminensis*, compared with mature, long-lived woody vegetation in the upper canopy layers. We nevertheless sampled the seedlings and saplings of shrubs and trees that were > 2 m tall at maturity if present within the ground-layer vegetation, as a measure of impacts of *T. fluminensis* invasion on forest recruitment. Non-native species were defined as those introduced to the Dandenong Ranges region of Victoria from other countries or other parts of Australia^54^.

Species abundance was quantified using a modified Braun-Blanquet scale of percentage foliage cover: 1, < 5% and a single individual; 2, < 5% and a few individuals; 3, < 5% with many individuals but uncommon across the plot; 4, < 5% and common across the plot; 5, < 5% and very abundant across the plot; 6, 5–20%; 7, 21–40%; 8, 41–60%; 9, 61–80%; 10, 81–100%^12^. Species were assigned to one of six growth form categories: (1) climbers (combining herbaceous twiners, scramblers and woody vines); (2) graminoids (combining grasses, sedges and rushes); (3) herbs, subdivided into tufted and spreading (i.e. rhizomatous and stoloniferous) forms; (4) ferns, subdivided into tree ferns (e.g. *Cyathea* spp. and *Dicksonia australis*) and ground-layer forms; (5) shrubs; (6) trees.

We derived information about five landscape-scale variables associated with anthropogenic development, using a combination of field measurements and spatial analysis on Google Earth (https://earth.google.com/web), adapted from procedures developed by Maisy et al.^51^. For each of the 83 plots, using the Ruler (Line) function in Google Earth, we calculated the minimum linear distance (m) from the nearest suburban garden (shown to be an important predictor of *T. fluminensis* invasion, including by dumping of garden waste^47,55^), footpath, road and forest edge (defined as the interface between the remnant native canopy and an anthropogenic landscape feature, which in many cases was a road or cluster of residential dwellings). Distance (m) to the water’s edge of the nearest stream was quantified in the field using a tape measure. While plots were initially selected based on a qualitative assessment of landscape context, we quantified these five landscape properties on a continuous scale to both objectively represent landscape context and explicitly test whether these contextual factors were influential (Table 4).

### Data analysis

All analyses were performed using R version 3.6.0^56^.

We fitted generalised linear mixed models to native species richness (number of native species per 4 m^2^ plot) and relative abundance (sum of % foliage cover abundance scores per plot) with Poisson error distributions and log-link functions. We fitted linear mixed models to native species diversity (Shannon–Weiner Diversity Index), as the data were normally distributed. We checked for multicollinearity amongst predictor variables and determined that *T. fluminensis* cover and volume were strongly correlated (*r* = 0.88, Fig. 1). Therefore, two separate models were used to test these effects on each response variable (referred to hereafter as the “trad cover model” and “trad volume model”). Importantly, no correlations were observed between variables representing “invasion” and those representing “landscape context”, meaning our targeted plot selection achieved its aim of a stratified design that could test the interactive effects of these two factors. Each native plant response variable was modelled as a function of both trad cover and volume, distance to forest edge, distance to stream, distance to road, distance to footpath, distance to garden, and the interaction of trad with each of the five distance measures (full model). All predictor variables were scaled to a mean of zero and a standard deviation of one prior to modelling to allow direct comparison of regression coefficients. We used Akaike’s Information Criterion corrected for small sample sizes (AICc) to rank subsets of the full model and determine the best (lowest AICc) and supported (ΔAICc < 2) models. We further sought to improve explanatory power by examining whether modelling trad (cover or volume) as a polynomial factor in supported models decreased AICc, in order to detect impact threshold effects (as per^12^).

Plots of residuals against fitted values, residual frequency histograms, quantile–quantile plots and residual variation box plots were examined to verify homogeneity and expected properties of residuals. Tests for overdispersion were undertaken to assess whether there was additional variance in the data than assumed by the error distributions. If models were overdispersed, as was the case for relative native abundance in both models, a random observation was included as a random effect to correct for the unexplained variance^57^. We used mixed models to account for spatial autocorrelation of plots with the inclusion of the landscape-level random effect “cluster” in all models. Model coefficients and 95% confident intervals were averaged and the predicted values calculated across the set of supported models (full averaging). Model predictions were the averaged predicted values calculated for each supported model, not a single prediction from the average coefficient. Any non-linear response curves were visually inspected for clear points of change in slope as indicators of impact thresholds.

Models were fitted using the ‘glmer’ function in the “lme4” package. Subsets of the full model were ranked using the ‘dredge’ function and coefficients averaged from supported models using the ‘model.avg’ function in the “MuMIn” package. Confidence intervals for averaged coefficients were estimated using the ‘confint’ function in the “stats” package, and predicted values were calculated using the ‘predictSE’ function in the “AICcmodavg” package.

We also fitted generalised linear mixed models to the native species richness and relative abundance of each of eight functional growth forms (climber, graminoids, ground ferns, tree ferns, spreading herbs, tufted herbs, shrubs, and trees). Model design (fixed and random effects), selection, averaging and checking follow as described above. Only “trad cover models” were utilised to test the main effects of *T. fluminensis* invasion on native plant growth forms as we were more interested in identifying patterns along the gradient of invasion establishment (from low to high cover) than in quantifying invasion dominance (the exponential development of volume at high cover) (Fig. 2).

### Consent for publication

All authors provide consent for publication.

## Supplementary Information

Supplementary Information 1.

Supplementary Information 2.

Supplementary Information 3.

## Data Availability

Data supporting our results is available as supplementary material, Appendix 3.
