# Supplementary figures and images for: Identifying thresholds in the impacts of an invasive groundcover on native vegetation
Source: Sci Rep. 2021 Oct 15;11:20512. doi: 10.1038/s41598-021-98667-5 (PMC8520009; doi:10.1038/s41598-021-98667-5)

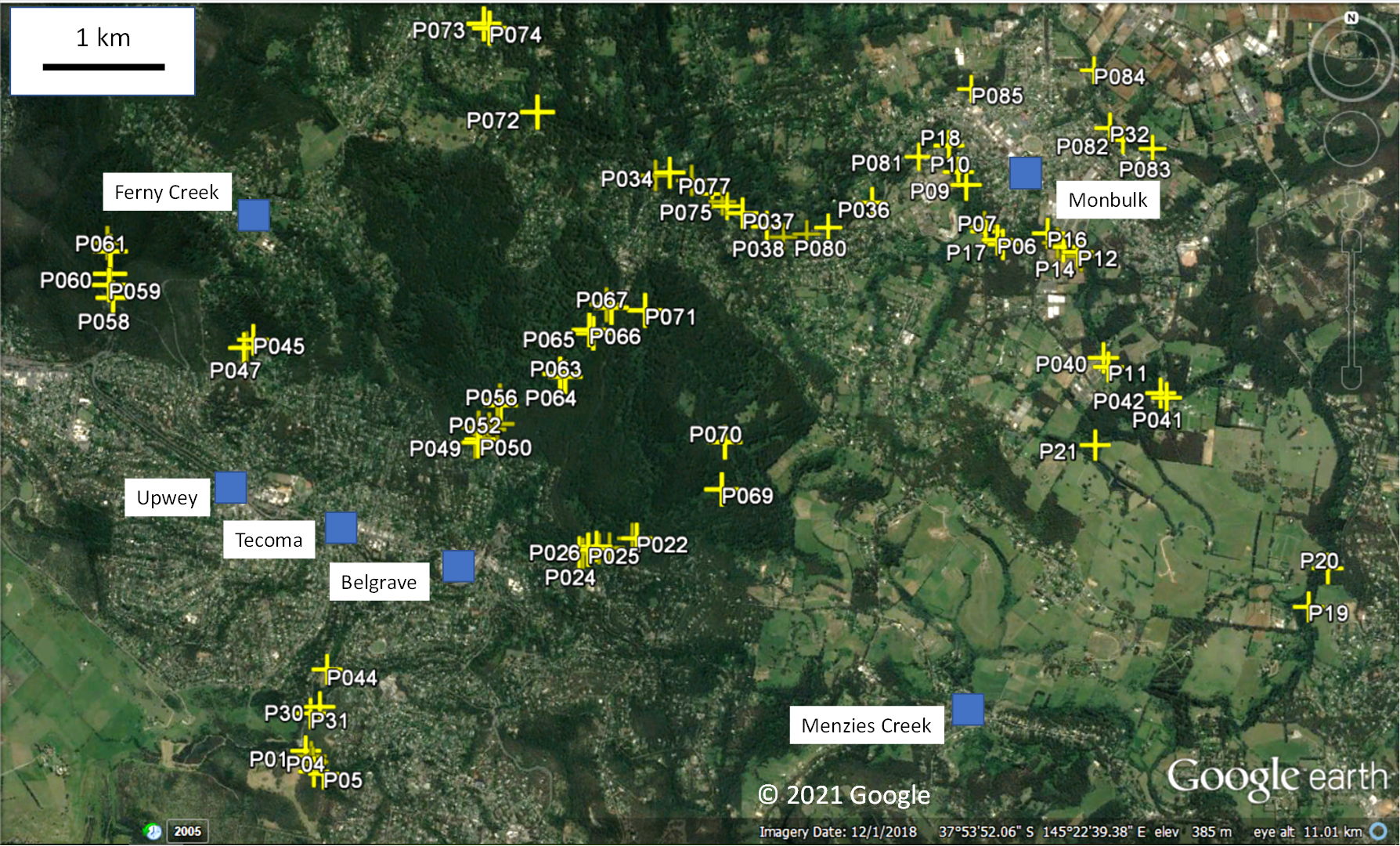

Supplement: Supplementary file 1 — Supplementary Information 1. [file 41598_2021_98667_MOESM1_ESM.png]
