# Supplementary material for: Identifying thresholds in the impacts of an invasive groundcover on native vegetation
Source: Sci Rep. 2021 Oct 15;11:20512. doi: 10.1038/s41598-021-98667-5 (PMC8520009; doi:10.1038/s41598-021-98667-5)
Supplement: Supplementary file 2 — Supplementary Information 2. [file 41598_2021_98667_MOESM2_ESM.pdf]

Appendix 2 – Photographic examples of Tradescantia fluminensis (wandering trad) infestations across the Dandenong Ranges region of Victoria, Australia

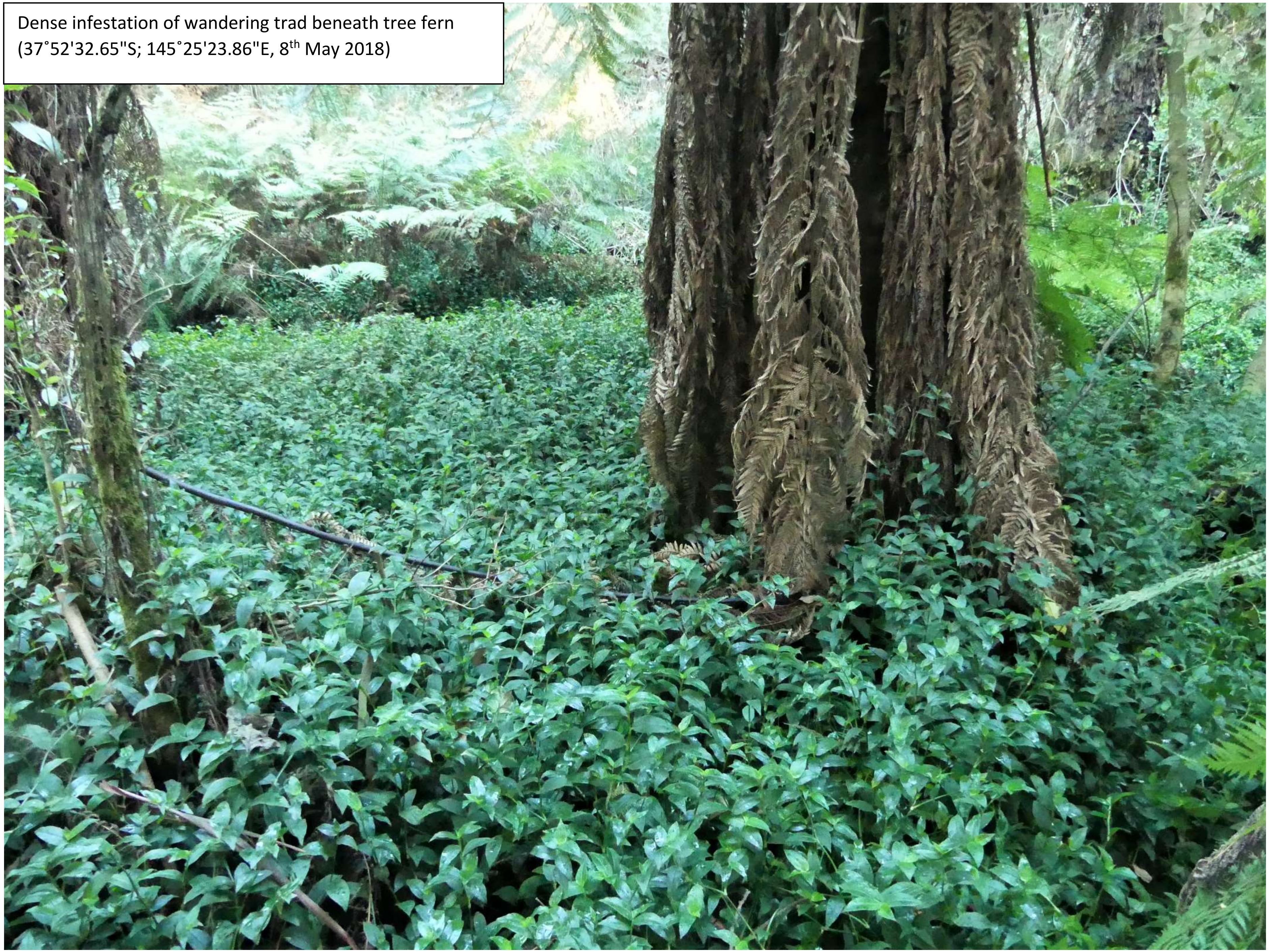

Dense infestation of wandering trad beneath tree fern
(37˚52'32.65"S; 145˚25'23.86"E, 8th May 2018)

Dense infestation of wandering trad beneath tree fern in association with a diverse array of native ground ferns (37˚53'35.56"S; 145˚25'8.42"E, 8th May 2018)

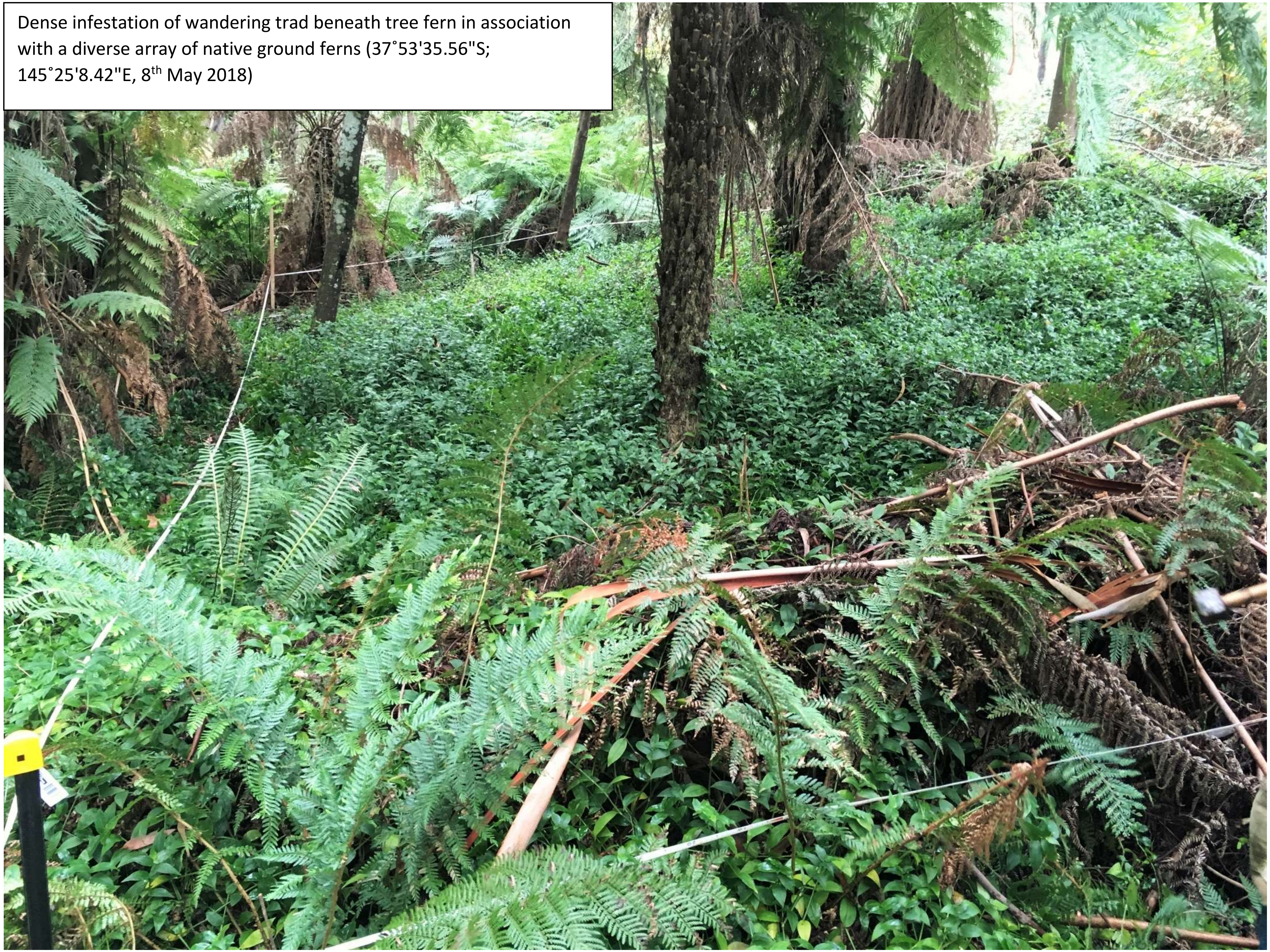

Dense infestation of wandering trad beneath tree ferns (*Cyathea* spp.) and canopy of *Pittosporum undulatum* (37˚55'2.34"S; 145˚20'21.51"E, 8th May 2018)

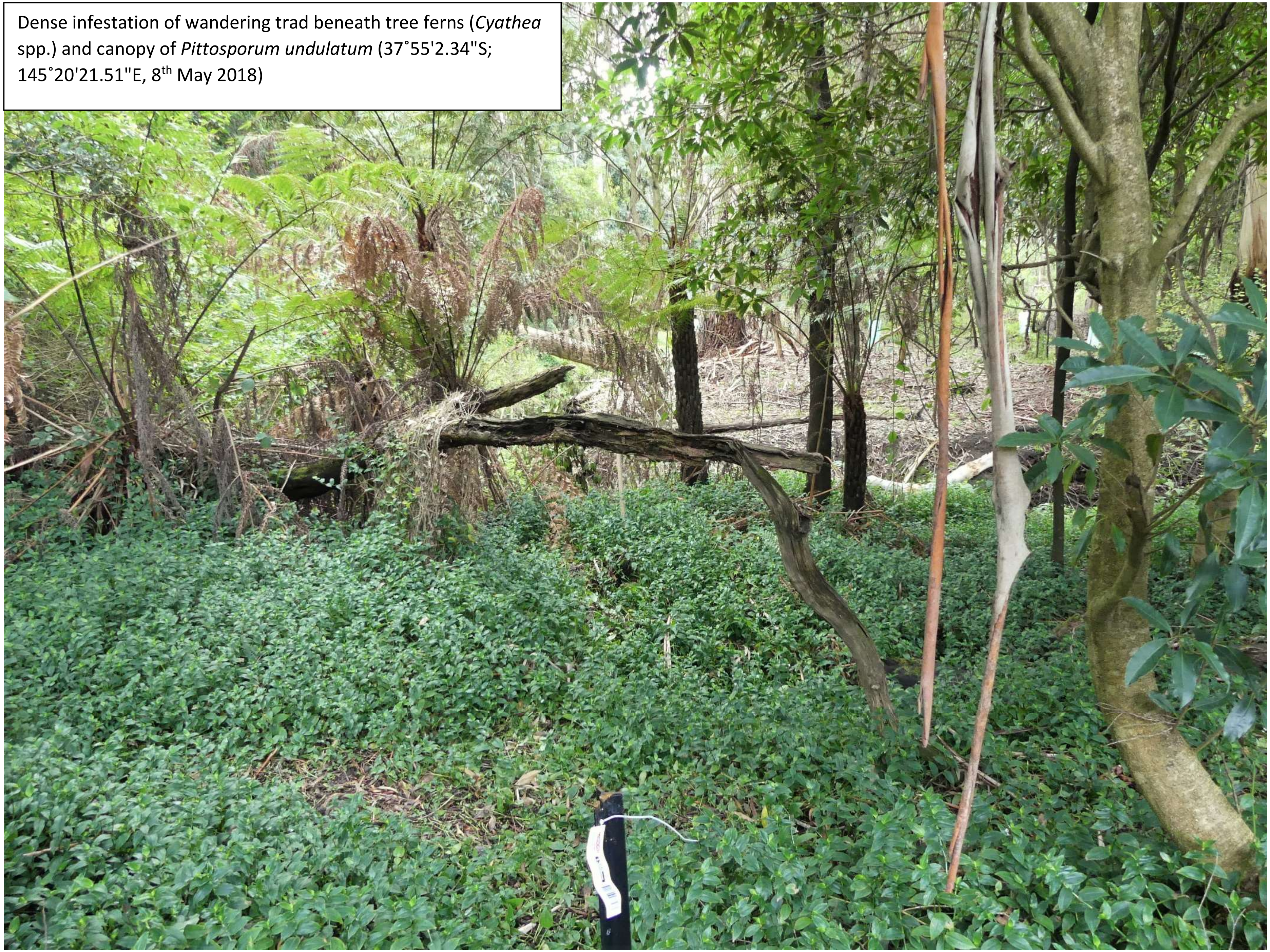

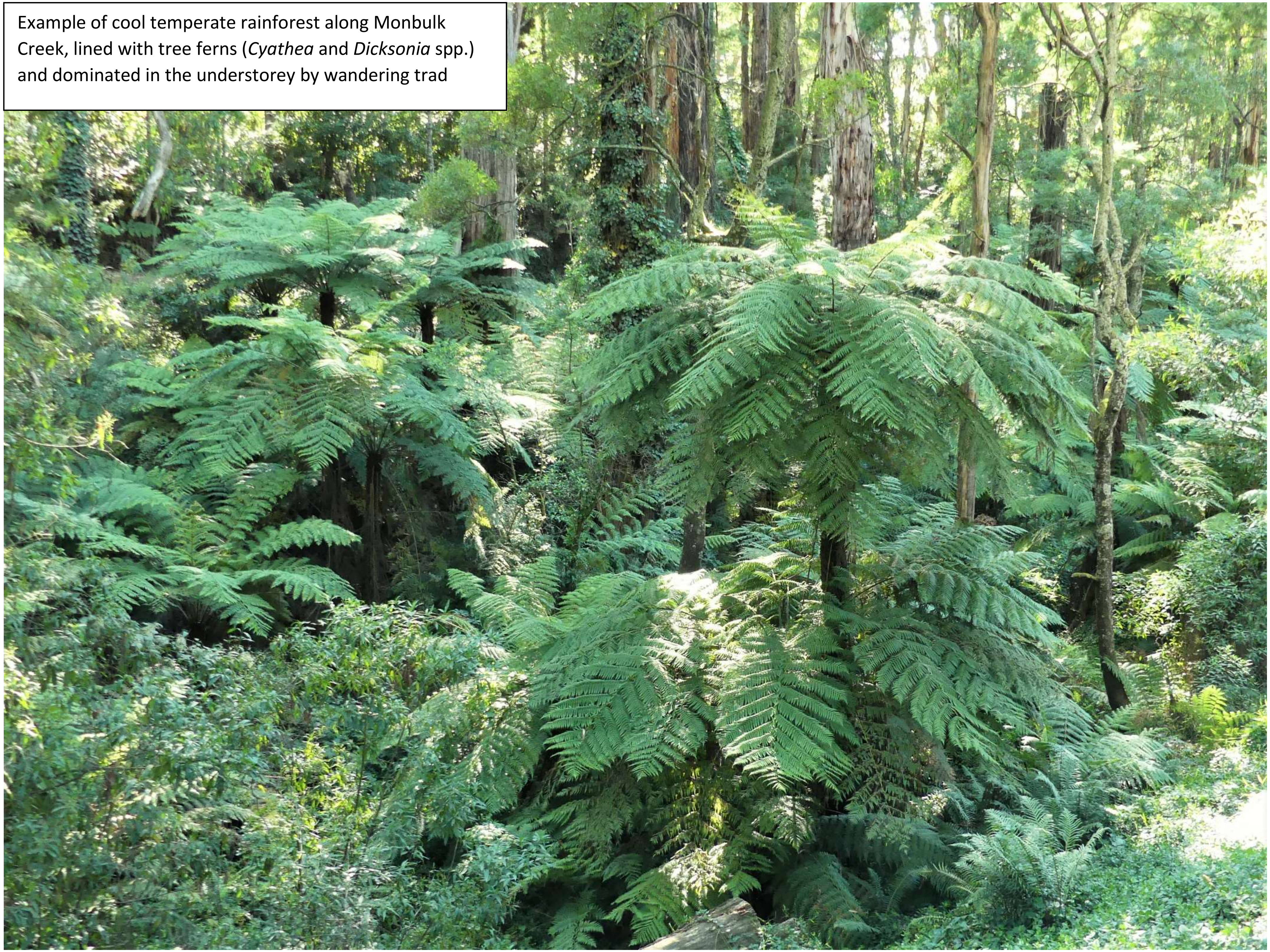

Example of cool temperate rainforest along Monbulk Creek, lined with tree ferns (*Cyathea* and *Dicksonia* spp.) and dominated in the understorey by wandering trad
